# *Streptomyces spiroverticillatus* induces poplar canker resistance against *Botryosphaeria dothidea*

**DOI:** 10.7717/peerj.20943

**Published:** 2026-04-17

**Authors:** Fengjiao Wang, Qiyue Ma, Fan Yang, He Mao, Liming Pan, Xiaoguang Zhang, Bo Yu, Limei Li, Chunyu Zhu

**Affiliations:** 1 Jilin Provincial Academy of Forestry Science, Changchun, Jilin, China; 2 School of Life Sciences, Liaoning University, Shenyang, Liaoning, China

**Keywords:** Poplar canker, *Streptomyces spiroverticillatus*, Induced resistance

## Abstract

*Botryosphaeria dothidea* is a destructive pathogen responsible for poplar canker, a disease that severely damages poplar trees by inducing stem lesions, growth inhibition, and eventual tree death, thereby causing substantial economic losses in forestry. Although conventional chemical fungicides are effective, their use poses significant environmental and health concerns, highlighting the need for safer biological control strategies. This study investigated the potential of *Streptomyces spiroverticillatus*, an antagonistic bacterium, to induce resistance in poplar against *B. dothidea*. Treatment with the cell-free supernatant (CFS) of *S. spiroverticillatus* markedly enhanced key defense-related physiological and biochemical responses in poplar leaves. Biochemical assays revealed that treated plants exhibited a 20% increase in total phenolics, a 37% rise in flavonoids, and a 40%–62% elevation in lignin content compared with controls. Furthermore, the activities of phenylalanine ammonia-lyase (PAL) and chitinase—enzymes critical for antimicrobial compound biosynthesis and fungal cell wall degradation—increased by 90% and 28%, respectively. Field trials confirmed the biocontrol efficacy of *S. spiroverticillatus*, reducing disease incidence by 61.17% (preventive) and 71.29% (curative), with corresponding disease reduction rates of 46.26% and 54.83%, respectively. These results outperformed carbendazim treatments, which showed preventive and curative efficacies of 49.97% and 50.04%, and disease reduction rates of 34.18% and 38.42%, respectively. These findings demonstrate that *S. spiroverticillatus* activates the innate defense mechanisms of poplar, stimulates the accumulation of antimicrobial metabolites and strengthens resistance against *B. dothidea*. This study identifies *S. spiroverticillatus* as a promising and environmentally sustainable biocontrol agent for the effective management of poplar canker.

## Introduction

Poplar canker, a fungal disease, significantly compromises the xylem health of poplar trees. Its characteristic symptoms include blister canker and dry rot canker, with the former being more prevalent. Initial infection is marked by the appearance of blister-like lesions on the trunk surface. These lesions subsequently expand, leading to tissue necrosis and, ultimately, potential tree mortality (Liu et al., 2005; Zueva et al., 1997). The disease is caused by the globally significant pathogen *Botryosphaeria dothidea* (Widelski et al., 2022). First reported in Beijing in 1955, poplar canker has since become prevalent across northern, northeastern, and southwestern China (Chen et al., 2024). While chemical fungicides have been the primary control method for decades (Tennakoon et al., 2019), growing concerns over their environmental and health impacts have accelerated the search for sustainable alternatives (Deng et al., 2024). Biological control, recognized as an eco-friendly strategy, is considered one of the most promising approaches for sustainable agriculture (Alam et al., 2010; Boukaew, Plubrukam & Prasertsan, 2013). The genus *Streptomyces*, the largest genus of actinomycetes, is a prolific source of biocontrol agents. *Streptomyces* spp. can inhibit phytopathogens through several biocontrol strategies, including the production of antifungal metabolites, secretion of cell wall-degrading enzymes (*e.g.*, chitinase and glucanase) (Torabi et al., 2019), synthesis of plant growth-promoting substances such as IAA (Oubaha et al., 2020), and induction of systemic resistance *via* defense-related signaling pathways (Nguyen et al., 2024). The practical significance of these metabolites is evidenced by successful field applications, such as *Streptomyces*-based formulations achieving up to 90.3% disease control in apple orchards (Dai et al., 2019). The efficacy of *Streptomyces* as a biocontrol agent is underscored by international studies. For instance, *Streptomyces* misionensis effectively controlled pistachio canker in Iran (Torabi et al., 2019), and plant growth-promoting *Streptomyces* from Morocco suppressed pea root rot while enhancing plant growth (Oubaha et al., 2020). These examples highlight the global relevance and dual benefit of pathogen inhibition and plant growth promotion. Induced resistance, a plant defense state triggered by external elicitors (Rao, Guan & Luo, 2000), involves complex mechanisms. These include structural changes like lignin and hydroxyproline-rich glycoprotein (HRGP) deposition and callose formation (Sun et al., 2021), physiological and biochemical processes such as phytoalexin accumulation and pathogenesis-related (PR) protein production, and molecular events where signal transduction activates defense genes. This study aims to determine whether the fermentation broth of *S. spiroverticillatus* can induce resistance in poplar against *B. dothidea*. We analyzed key defense-related biochemical indicators—including total phenolics, flavonoids, lignin, and the activities of phenylalanine ammonia-lyase (PAL) and chitinase—to evaluate the induced resistance potential and elucidate the underlying physiological mechanisms.

## Materials and Methods

### Strains and culture conditions

*Streptomyces spiroverticillatus* was isolated from soil sampled at the Hengshan Protection Station, Changbai Mountain National Nature Reserve, Jilin Province, China, and identified by whole-genome sequencing (GenBank: MN636764). The isolation and purification procedures were performed following the method described by Zhao, Wang & Lv (2021). The pathogen *Botryosphaeria dothidea*, the causal agent of poplar canker, was provided and preserved by the Jilin Provincial Academy of Forestry Sciences, and its identity was confirmed through multigene phylogenetic analysis based on ITS, tef1, and tub2 loci.

### Preparation of fermentation broth

A seed culture of *S. spiroverticillatus* was prepared in YMG broth (28 °C, 180 rpm, 2 d) (Shi et al., 2024), followed by transfer into fresh medium (1% inoculum) for fermentation under identical conditions for 7 d. The fermentation broth was centrifuged at 10,000 rpm for 15 min, and the supernatant was sterilized by filtration through a 0.22 µm membrane before being used in subsequent experiments, which was applied at a 50-fold dilution in subsequent assays.

### Field experimental design and treatments

A total of 1,500 four-year-old poplars (*Populus* pseudo-simonii × *P. nigra*) were grown under natural conditions across approximately 1.8 ha at the experimental base in Yitong, Jilin Province. For both the protective and curative assays, the trees were randomly allocated into the following groups, each comprising 300 trees: (i) *S. spiroverticillatus* fermentation broth, (ii) carbendazim, and (iii) an uninoculated sterile culture medium control . The same control group (300 trees) was shared between the protective and curative experimental arms. Thus, the trial consisted of 300 trees per treatment per assay type, with a common control. The treatments were applied as follows: T1: *S. spiroverticillatus*: sterile fermentation broth (50-fold dilution). T2: Carbendazim: 800-fold dilution. Control: uninoculated sterile culture medium. All treatments were applied using trunk-directed sprays to ensure uniform coverage.

### Pathogen inoculation and sampling

Selected trees were inoculated by making a one cm wound at the trunk base with a scalpel, sterilizing with an alcohol blast burner for 10 s, and placing a seven mm mycelial plug of *B. dothidea* on the wound. The site was moistened with sterile water-soaked cotton and wrapped with sealing film. After 7 days, wraps were removed, disease development was recorded, and the presence of conidiophores and conidial horns was confirmed. Leaf sampling started 3 days after treatment and was repeated every 3 days for a total of seven sessions. Fresh leaves were collected, midribs removed, cut into 2–3 mm pieces, and 0.2 g samples were flash-frozen in liquid nitrogen, stored at −80 °C (Chen et al., 2025).

### Treatment regimes for bioassay

Two types of bioassays were conducted: Preventive (pre-inoculation) assay: solutions were applied three times at 72-hour intervals before pathogen inoculation. Curative (post-inoculation) assay: applications started 72 h after inoculation, also performed three times at 72-hour intervals. In both assays, 50 mL of solution was applied above and below the inoculation site within a 20 cm range. Controls received unfermented sterile medium in the same manner.

### Disease assessment

Inoculated plants were assessed for disease incidence at 15 d post-inoculation. The disease index grading standard adopted in this experiment was in line with the provisions of Technical Regulations of Integrated Management of Valsa sordida (DB22/T 1768-2013) issued by the Jilin Provincial Administration for Quality and Technical Supervision (Zhang, 2013).

\begin{eqnarray*}\mathit{Disease~ rate}= \frac{Number~of~infected~trees}{Total~number~of~trees} \times 100\% \end{eqnarray*}

\begin{eqnarray*}\mathit{Disease~ index}\nonumber\\\displaystyle  =\sum \frac{\mathit{Number~ of~ diseased~ plants~ at~ each~ level}\times \mathit{Representative~ values~ at~ all~ levels}}{\mathit{Total~ number~ of~ plants~ surveyed}\times \mathit{Highest~ representative~ value}} \times 100 \end{eqnarray*}

\begin{eqnarray*}\mathit{Effectiveness~ of~ prevention~ and~ curation}\nonumber\\\displaystyle  = \frac{\mathit{Control~ disease~ index}-\mathit{Treatment~ disease~ index}}{\mathit{Control~ disease~ index}} \times 100\% \end{eqnarray*}

\begin{eqnarray*}\mathit{Disease~ rate~ decrease}= \frac{\mathit{Control~ disease~ rate}-\mathit{Treatment~ disease~ rate}}{\mathit{Control~ disease~ rate}} \times 100\%. \end{eqnarray*}

### Effect of the fermentation broth on the metabolic pathway of phenylalanine in *Populus*

#### Determination of the total phenolic contents of poplar

Poplar leaf samples (0.1 g) were transferred into 10 mL centrifuge tubes and mixed with 10 mL of extraction solution consisting of HCl:methanol (1:100, v/v). After incubation at 4 °C for 24 h, 0.4 mL of the supernatant was combined with four mL of the extraction solution, diluted tenfold, and thoroughly mixed. Absorbance was recorded at 280 nm using a UV spectrophotometer (Waters, Milford, MA, USA), and the total content of phenolics was calculated by a standard curve. Each treatment was replicated three times (Wang, 2023).

\begin{eqnarray*}\mathit{Total~ phenolic~ content~ (mg/g)}\nonumber\\\displaystyle  = \frac{\mathit{Total~ phenol~ content~ after~ dilution~ (mg/mL)}\times \mathit{Volume~ of~ Extraction~ Solution~ (mL)}\times \mathit{Dilution~ times}}{\mathit{Fresh~ weight~ (g)}} . \end{eqnarray*}

#### Determination of the flavonoid content of poplar

The contents of flavonoids were determined by taking 0.02 g of poplar leaves and placing them in a 10 mL centrifuge tube. Each sample was mixed with 10 mL of 75% aqueous ethanol and was subsequently incubated in a 70 °C water bath for 20 min. Following this treatment, samples were stored at 4 °C for 24 h. A volume of 0.3 mL of 5% sodium nitrite was added. The mixture was thoroughly mixed. After 6 min of stationary incubation, 0.3 mL of 10% aluminum nitrate was added.The resulting mixture was thoroughly vortexed and allowed to stand for 6 min. Thereafter, four mL of 1 mol/L sodium hydroxide solution was added, followed by the addition of 0.4 mL distilled water to bring the final volume to 10 mL. After a further incubation period of 20 min, absorbance was measured at 510 nm. Flavonoid content was determined based on a rutin standard curve prepared in anhydrous ethanol. All treatments were performed in triplicate (Zheng et al., 2023).

\begin{eqnarray*}\mathit{Flavonoid~ content~ (mg/g)}\nonumber\\\displaystyle  = \frac{\mathit{Regression~ equation~ flavonoid~ content~ (mg/mL)}\times \mathit{Volume~ of~ Extraction~ Solution~ (mL)}\times \mathit{Dilution~ times}}{\mathit{Fresh~ weight~ (g)}} \end{eqnarray*}

#### Determination of the lignin content of poplar

The lignin assay kit was purchased from Suzhou Michy Biomedical Technology Co., Ltd, following the instructions in the kit. Each treatment was replicated three times.

\begin{eqnarray*}\mathit{Lignin~ content~ (mg/g~ Dry~ weight)}= \frac{(\Delta A-0.0068)}{0.002} \times 50. \end{eqnarray*}

#### Determination of phenylalanine ammonia-lyase PAL activity in poplar

Phenylalanine ammonia-lyase (PAL) was extracted by adding one mL of 0.1 mol/L boric acid buffer (pH 8.8) to 0.1 g of poplar leaves and homogenizing them by grinding with liquid nitrogen in a ball mill. The homogenate was centrifuged (10,000 rpm, 4 °C, 15 min), and the supernatant collected as crude enzyme extract was stored at −80 °C until analysis. For the enzyme assay, 300  µL of crude extract was combined with 2.9 mL of boric acid buffer (pH 8.8) and one mL of 0.02 mol/L L-phenylalanine to a final volume of four mL. The solutions were thoroughly mixed and incubated in a 30 °C water bath for 60 min. A volume of 0.2 mL of 6 mol/L HCl was added to terminate the reaction. If there was precipitate after termination, the reaction mixture was centrifuged at 10,000 rpm for 5 min at 4 °C to remove it. The control utilized the boric acid buffer, and the absorbance values of the reaction solution were measured at 290 nm with the control adjusted to zero. The amount of enzyme required to increase the absorbance value of the reaction system at 290 nm by 0.01 per min was considered to be 1 unit (U) of activity. Each treatment was replicated three times (Chen et al., 2021).

\begin{eqnarray*}\mathit{PAL~ Active~ [U/(gFW.min)]}= \frac{\mathit{A}\times \mathit{Vt}\times \mathit{v}}{\mathit{0.01}\times \mathit{Vs}\times \mathit{FW}\times \mathit{t}} . \end{eqnarray*}

Vt: total volume of enzyme solution (mL); FW: fresh weight of the sample (g); Vs: the amount of enzyme solution taken at the time of determination (mL); v: total volume of reaction solution (mL); t: reaction time (min).

### Effect of fermentation broth on the activity of chitinase in poplars

A volume of one mL of extraction solution was added to 0.1 g of poplar leaves, and the tissue was homogenized in liquid nitrogen using a ball mill. The homogenate was centrifuged at 10,000 g at 4 °C for 20 min. The supernatant was the crude enzyme solution, which was stored at −20 °C for later use. The CHI assay kit was purchased from Suzhou Michy Biomedical Technology Co., Ltd, following the instructions in the kit. Each treatment was replicated three times.

\begin{eqnarray*}\mathit{Chitinase~ activity~ (mg/gFW.h)}= \frac{\mathit{7.799}\times (\Delta A+\mathit{0.2753})}{\mathit{Fresh~ weight~ (g)}} . \end{eqnarray*}

### Data analysis

All experiments in this study were conducted with three independent replicates. Data are presented as the mean ± standard error (SE). Graphs were generated using Origin 2018. Statistical analysis, including one-way analysis of variance (ANOVA), was performed with IBM SPSS Statistics 25.0. Mean values were compared using Duncan’s multiple range test, and differences were considered statistically significant at *P* < 0.05.

## Results and Analysis

### Field control efficacy of *S. spiroverticillatus* against poplar canker

The field efficacy of *S. spiroverticillatus* fermentation broth against poplar canker was evaluated in both protective and curative applications (Tables 1 and 2). In the protective assay (Table 1), treatment with *S. spiroverticillatus* resulted in a disease incidence of 51.32% and a disease index of 25.31, representing a disease reduction of 46.26%. These values were significantly lower than those in the carbendazim-treated group (62.82%, 32.55, and 34.18% reduction, respectively; *P* < 0.05). The preventive efficacy of *S. spiroverticillatus* reached 61.17%, significantly outperforming carbendazim (49.97%). In the curative assay (Table 2), *S. spiroverticillatus* demonstrated even stronger activity after pathogen establishment. It achieved a disease incidence of 43.07%, a disease index of 18.73, and a disease reduction of 54.83%, all of which were significantly superior to the results of the carbendazim treatment (58.66%, 32.51, and 38.42% reduction, respectively). The therapeutic efficacy was calculated at 71.29%, which was significantly higher than that of carbendazim (50.04%). Visual observations at 15 days post-inoculation confirmed these quantitative results: lesions expanded markedly with prolific conidiation in control trees, whereas no lesion progression was observed in trees treated with *S. spiroverticillatus* fermentation broth.

**Table 1:** Protective efficacy of *S. spiroverticillatus* fermentation broth against poplar canker under field conditions (*n* = 900).

Treatment	Disease rate (%)	Disease index	Disease rate decrease (%)	Prevention (%)
*S. spiroverticillatus*	51.32 ± 7.70^bc^	25.31 ± 3.52^b^	46.26 ± 4.81^a^	61.17 ± 3.12^a^
Carbendazim	62.82 ± 8.33^b^	32.55 ± 2.46^b^	34.18 ± 4.76^b^	49.97 ± 2.13^b^
Control	95.07 ± 7.83^a^	65.03 ± 3.27^a^	–	–

**Notes.**

Data in the table are the mean ± SD. There was a significant difference at *P* < 0.05.

Different lowercase letters in the same column indicate significant differences at the *P* < 0.05 level by Duncan’s new multiple range test, respectively.

**Table 2:** Curative efficacy of *S. spiroverticillatus* fermentation broth against poplar canker under field conditions (*n* = 900).

Treatment	Disease rate (%)	Disease index	Disease rate decrease (%)	Curation (%)
*S. spiroverticillatus*	43.07 ± 7.22^c^	18.73 ± 4.53^c^	54.83 ± 4.46^a^	71.29 ± 3.28^a^
Carbendazim	58.66 ± 8.07^bc^	32.51 ± 2.62^b^	38.42 ± 4.84^b^	50.04 ± 1.18^b^
Control	95.07 ± 7.83^a^	65.03 ± 3.27^a^	–	–

**Notes.**

Data in the table are the mean ± SD. There was a significant difference at *P* < 0.05.

Different lowercase letters in the same column indicate significant differences at the *P* < 0.05 level by Duncan’s new multiple range test, respectively.

### Effect of the metabolic pathway of phenylalanine in poplars

#### Total phenolic content in poplar leaves

**Figure 1:**
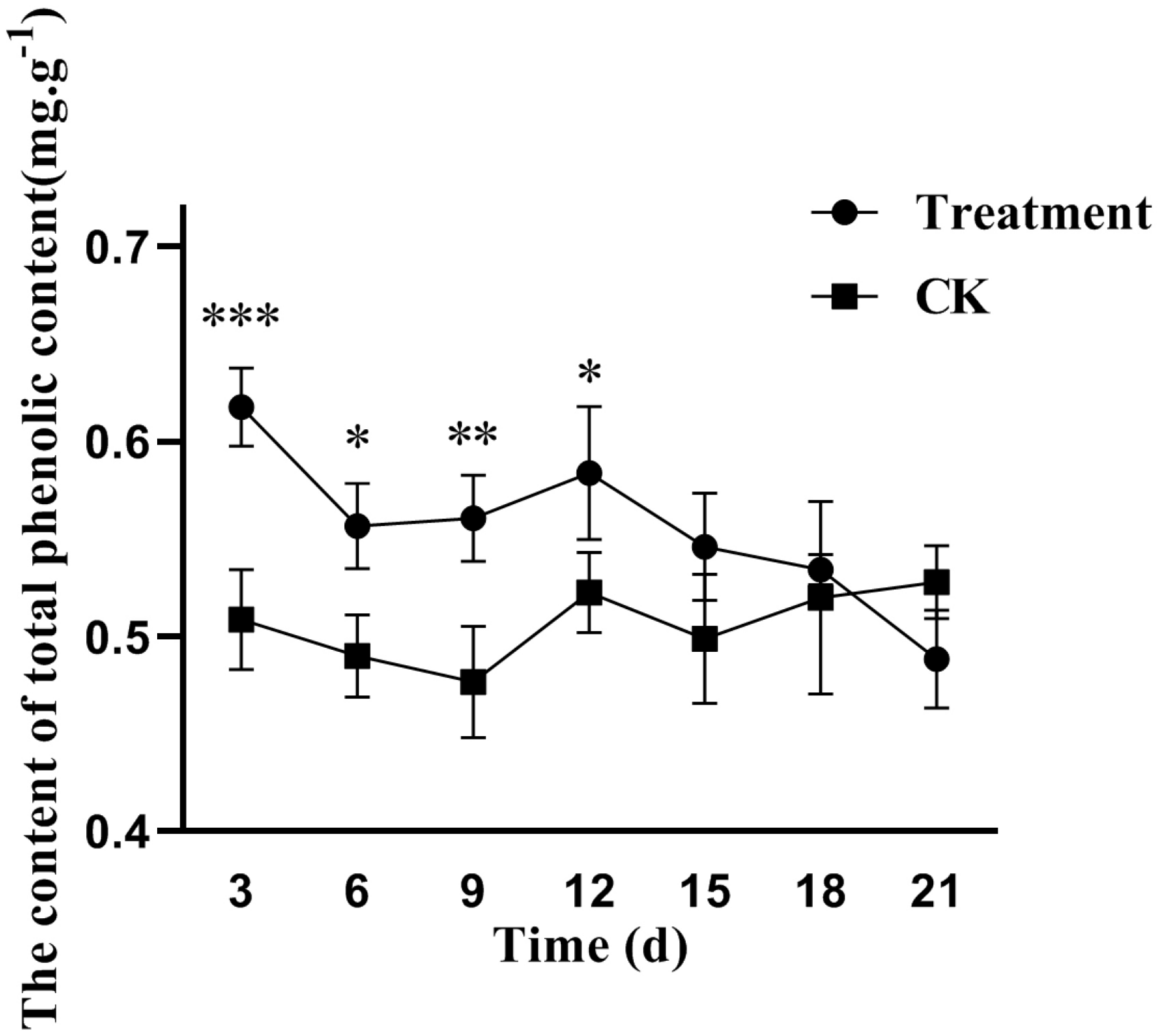
Effects of *S. spiroverticillatus* fermentation broth on the content of total phenolics in poplar leaves. Note: All values are expressed on a fresh weight basis (FW).Differences between samples were determined by a two-tailed Student’s *t*-test at *P* < 0.05. *0.01 < *P* < 0.05; **0.01 < *P* < 0.001; ***P < 0.001.

The synthesis of most phenolics in higher plants is initiated by phenylalanine. The phenolics include coumarins, phenylpropanoids, anthocyanins, tannins, benzoic acid derivatives, flavonoids and isoflavones, which are collectively known as phenylpropanoids. Phenylalanine ammonia-lyase (PAL) is at the intersection of primary and secondary metabolism and is the key enzyme that catalyzes the formation of phenolics (Deng et al., 2024). After 12 days of treatment with the fermentation broth of the biocontrol strain *S. spiroverticillatus*, the total phenolic content of poplar leaves was generally higher than that of the control group and the difference was statistically significant (*P* < 0.05, Fig. 1). The total phenolic content of the treated group showed an increase of 20% compared to the control group at 3 d. This indicates that the fermentation broth of the biocontrol *S. spiroverticillatus* can significantly induce the accumulation of phenolics in poplar leaves.

#### Flavonoid content in poplar leaves

**Figure 2:**
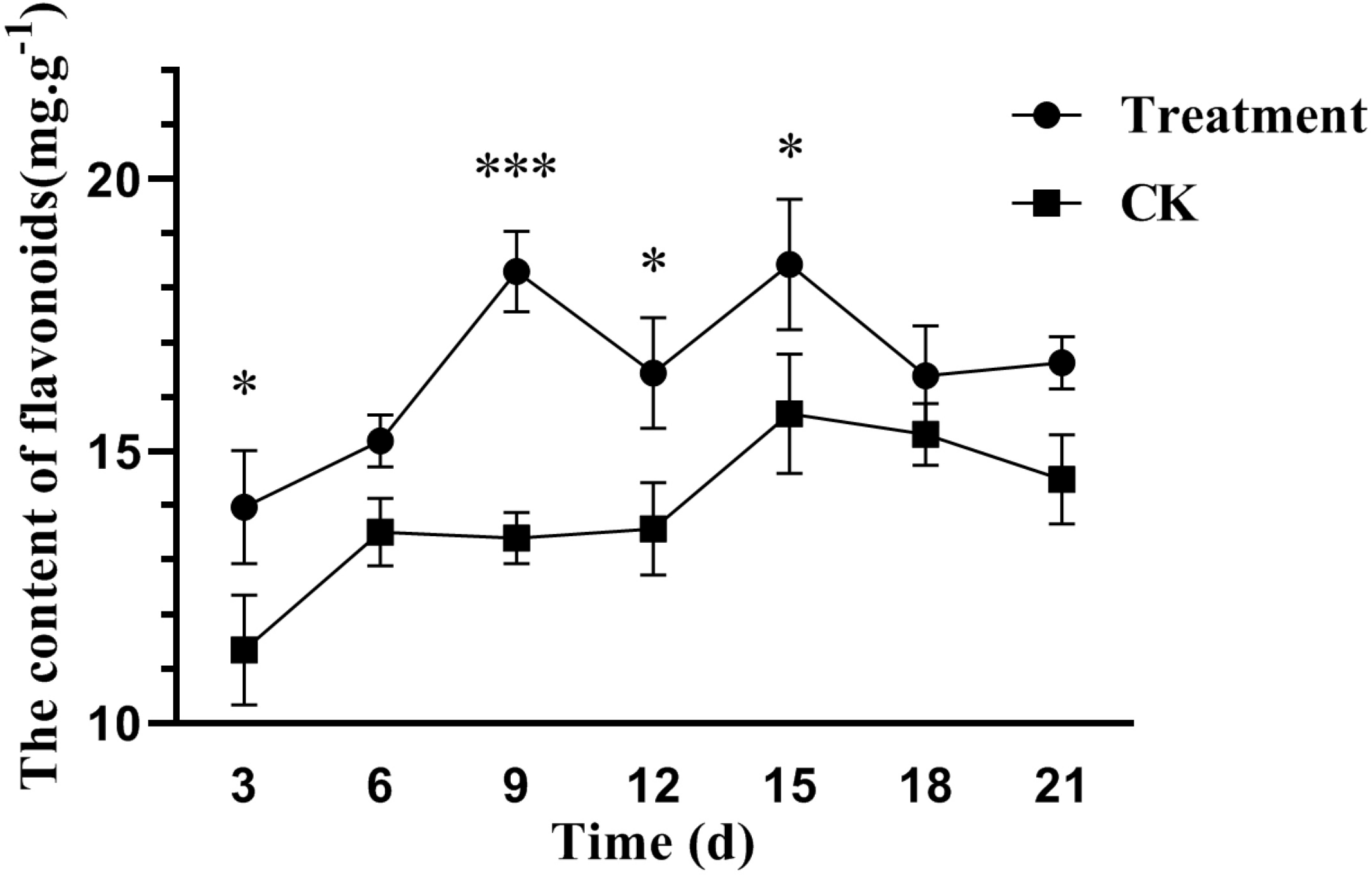
Effects of *S. spiroverticillatus* fermentation broth on the content of flavonoids in poplar leaves. Note: All values are expressed on a fresh weight basis (FW). Differences between samples were determined by a two-tailed Student’s *t*-test at *P* < 0.05. *0.01 < *P* < 0.05; **0.01 < *P* < 0.001; ***P < 0.001.

Flavonoids, one of the most abundant groups of plant phenolic compounds, perform biological functions such as pigment formation and defense. As shown in Fig. 2, the flavonoid content in poplar leaves increased significantly at 3, 9, 12, and 15 days after treatment with the fermentation broth of the biocontrol strain *S. spiroverticillatus*. At 9 days, the flavonoid content in the treated leaves was 37% higher than that in the control. This indicates that the fermentation broth can significantly induce the accumulation of flavonoids in poplar leaves.

#### Lignin content in poplar leaves

**Figure 3:**
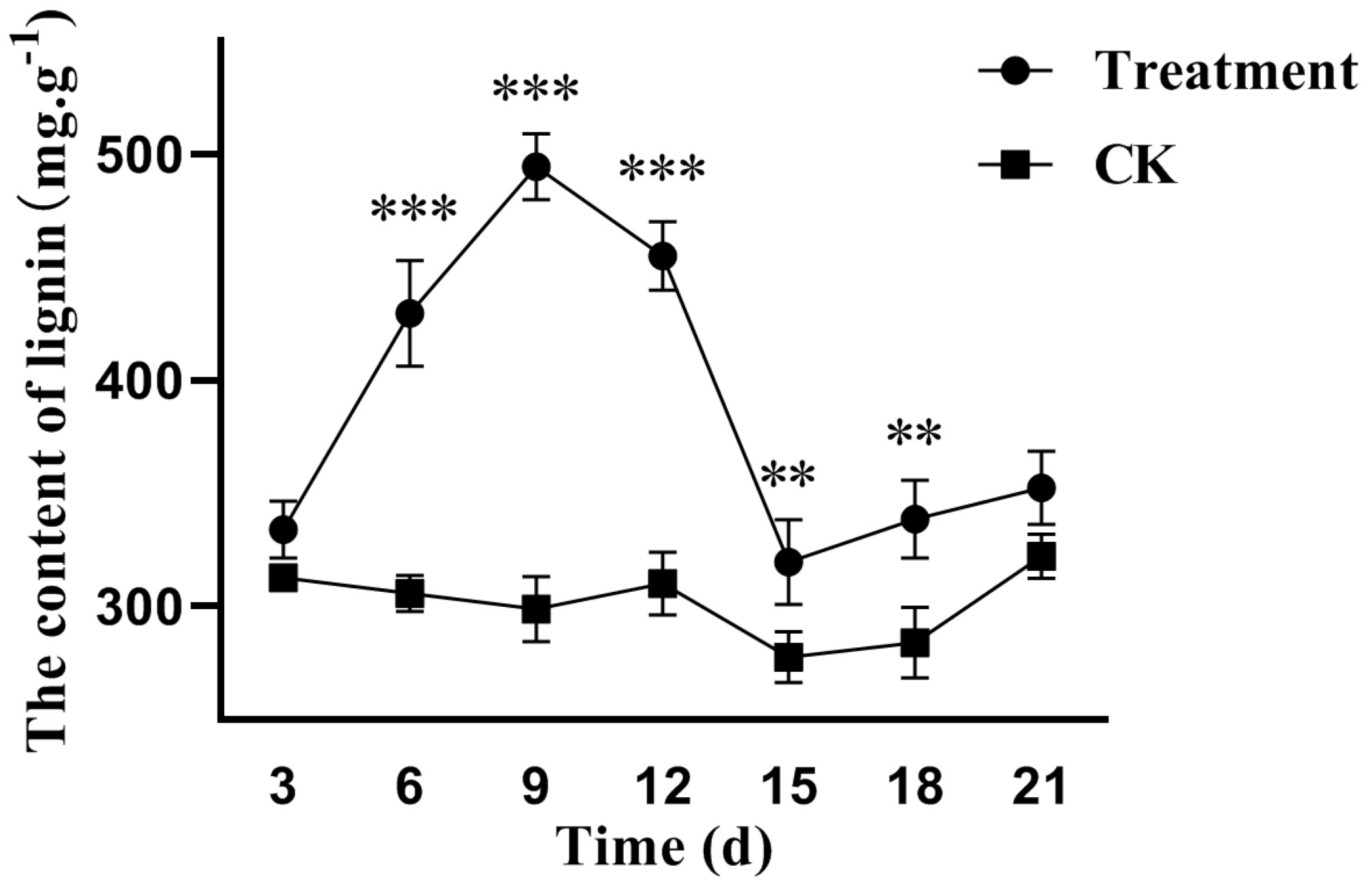
Effects of *S. spiroverticillatus* fermentation broth on the content of lignin in poplar leaves. Note: Differences between samples were determined by a two-tailed Student’s *t*-test at *P* < 0.05. *0.01 < *P* < 0.05; **0.01 < *P* < 0.001; ***P < 0.001.

Lignin, a complex phenolic macromolecule, is a key component of water transport in plants. The lignin content in poplar leaves was significantly higher after treatment with the fermentation broth of the biocontrol strain *S. spiroverticillatus* (Fig. 3). Significant increases were observed from 6 to 18 days after treatment. During the peak period, lignin content increased by 40%, 62%, and 43% at 6, 9, and 12 days, respectively, compared to the control. These results indicate that the fermentation broth significantly induces lignin accumulation in poplar leaves.

#### Activity of the poplar leaf phenylalanine ammonia-lyase

**Figure 4:**
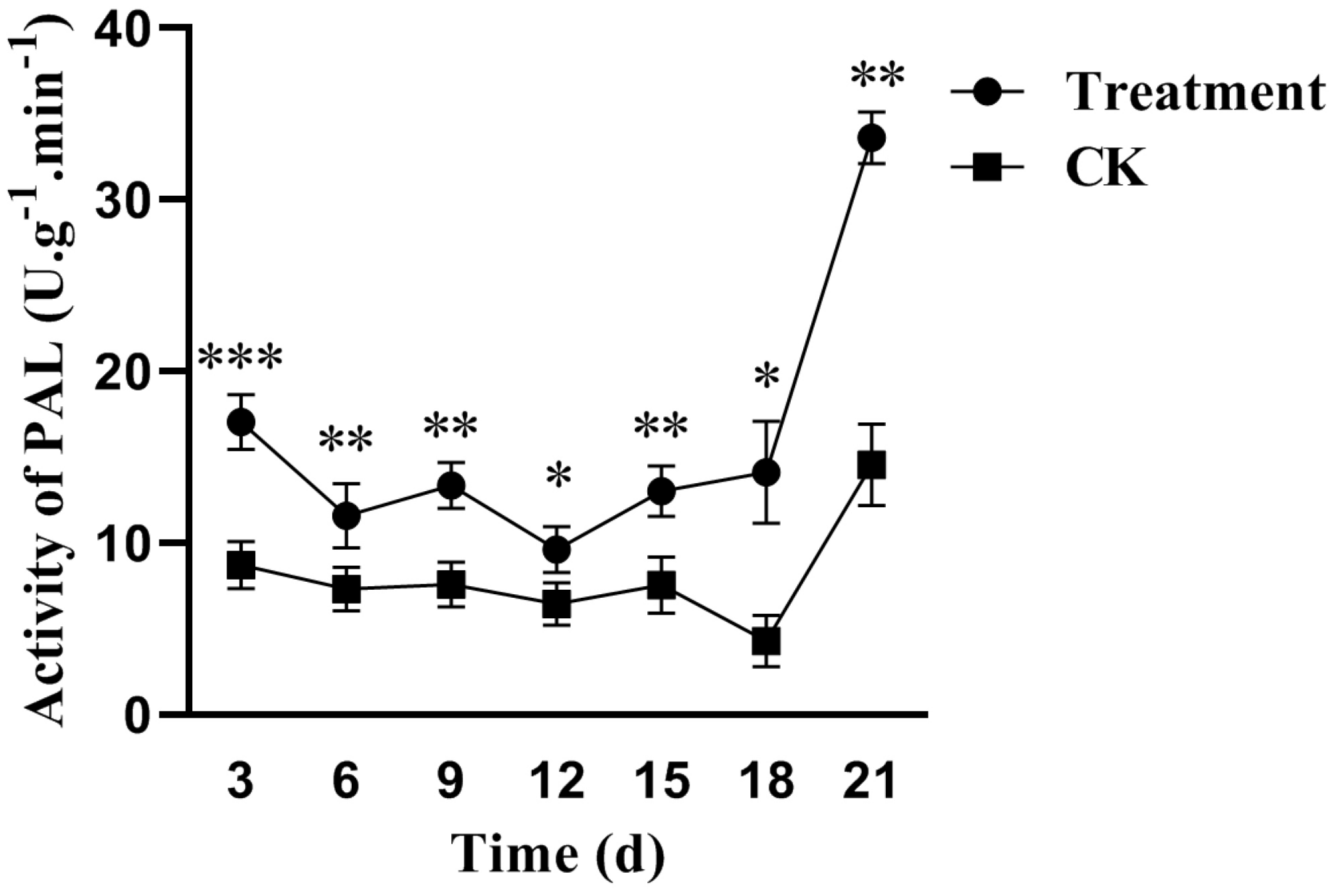
Effects of *S. spiroverticillatus* fermentation broth on PAL in poplar leaves. Note: All values are expressed on a fresh weight basis (FW). Differences between samples were determined by a two-tailed Student’s *t*-test at *P* < 0.05. *0.01 < *P* < 0.05; **0.01 < *P* < 0.001; ***P < 0.001.

Phenylalanine ammonia-lyase (PAL) is a widely studied enzyme in the secondary metabolism of plants, and it has an important regulatory role in the formation of many phenolic substances. The PAL activity of poplar leaves was significantly higher than that of the control group within 21 d after treatment with the fermentation broth of *S. spiroverticillatus*, and the difference was highly significant, particularly on day 3, which showed an increase of 90% compared to that of the control group (Fig. 4). The formation of various phenolic substances *via* the phenylalanine metabolism pathway was significantly enhanced. This enhanced PAL activity indicates that the fermentation broth of the biocontrol strain *S. spiroverticillatus* can significantly induce the production and accumulation of diverse phenolic substances in poplar. These antimicrobial secondary metabolites contribute to the tree’s resistance against pathogenic fungi.

### Effect of fermentation broth on the activity of poplar leaf chitinase during disease development

**Figure 5:**
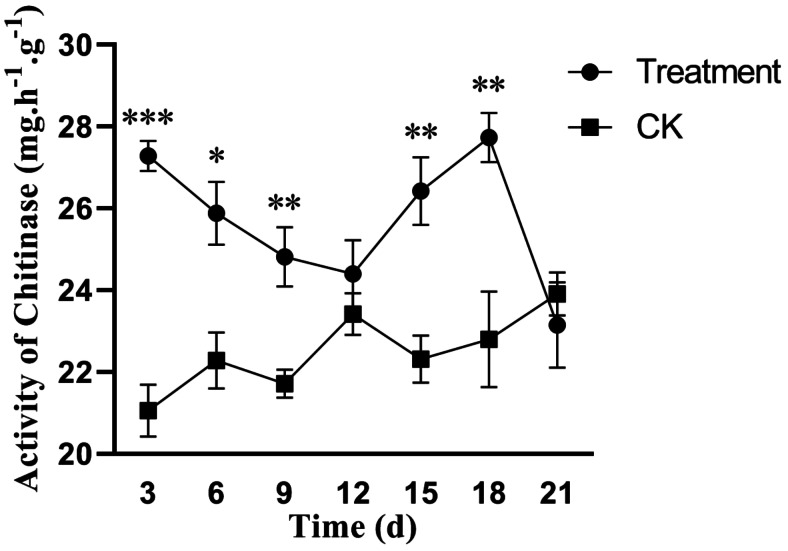
Effects of *S. spiroverticillatus* fermentation broth on the activity of chitinase in poplar leaves. Note: All values are expressed on a fresh weight basis (FW). Differences between samples were determined by a two-tailed Student’s *t*-test at *P* < 0.05. *0.01 < *P* < 0.05; **0.01 < *P* < 0.001; ***P < 0.001.

Plants deploy a defense response involving the production of hydrolases, such as glucanase and chitinase, which degrade fungal cell walls. Chitin, the primary component of fungal cell walls, is a key target of chitinase. Thus, enhanced chitinase activity can bolster plant resistance to pathogen invasion. As shown in Fig. 5, chitinase activity was significantly higher in poplar trees treated with the biocontrol strain *S. spiroverticillatus*. At 3 days post-treatment, the activity was 28% higher than that in the control. This result indicates that the fermentation broth of *S. spiroverticillatus* can induce chitinase activity in poplar.

## Discussion

Poplar canker, a severe fungal disease, often elicits enhanced host defense responses upon infection—an observation suggestive of a primed resistance state (Song & Wu, 2011; Li, Shu & Liu, 2002). This induced resistance mechanism resembles that observed in mycorrhizal plants, which display accelerated defense activation, leading to the rapid accumulation of antimicrobial compounds and improved pathogen resistance (Lebeis et al., 2015). In this study, we observed a significant increase in PAL activity in poplar leaves following treatment with *S. spiroverticillatus*, which is closely associated with the activation of the phenylpropanoid metabolism pathway. Previous research has indicated that PAL, as a key rate-limiting enzyme in this pathway, exhibits a rapid increase in activity upon pathogen infection, thereby enhancing disease resistance. Our data further support the importance of this mechanism in the interaction between poplar and canker disease (Hu, You & Pan, 2020; Shi, Qiang & Zhang, 2023). Previous studies have shown that PAL expression is induced during the early stages of infection, whereas feedback regulatory mechanisms later attenuate its activity (Bamneshin et al., 2022). In this study, treatment with *S. spiroverticillatus* resulted in a marked elevation of PAL activity in poplar leaves, indicating the activation of the phenylpropanoid metabolism. This activation led to the subsequent accumulation of phenolic compounds, which play crucial roles in plant structural defense and antimicrobial activity. Chitinase plays a crucial role in plant defense by degrading chitin, a major component of fungal cell walls (Peng et al., 2016). In this study, the significant increase in chitinase activity observed in *S. spiroverticillatus*-treated poplars (Fig  5) suggests that this enzyme contributes to the suppression of *B. dothidea* through cell wall disruption and inhibition of hyphal growth (Sytwala, Günther & Melzig, 2015). Our results demonstrated a significant increase in chitinase activity in *S. spiroverticillatus*—treated poplars, confirming its role in reinforcing host resistance against *B. dothidea*. *B. dothidea* exhibits a broad host range, causing serious canker diseases in crops such as pecan (*Carya illinoensis*) (Cheng & Xu, 2017) and blueberry (*Vaccinium* sect. *Cyanococcus*) (Kang et al., 2019). This broad host range and economic impact highlight the need for effective control measures. While previous research has identified antagonistic strains like (*Bacillus amyloliquefaciens* with inhibitory effects on this pathogen (Chen et al., 2015), there remains a need to discover highly effective, environmentally compatible biocontrol agents. The strain *S. spiroverticillatus* shows considerable potential in this regard. It was originally isolated from soil in the Hengshan Station of Changbai Mountain, China, and shares morphological characteristics with *Streptomyces* RB72T isolated from Alabama, USA (Farris et al., 2011). Secondary metabolites of *S. spiroverticillatus* include several novel compounds with reported antifungal activities (Liu et al., 2023). The current study, together with earlier field experiments (Chen et al., 2021; Ghorbel et al., 2014), supports that *S. spiroverticillatus* fermentation products can suppress *B. dothidea* and trigger host defense responses.

Nevertheless, this study has certain limitations. The experiments were conducted at a single site within one growing season. Variability due to environmental factors such as temperature, humidity, and soil type was not assessed. Future research should therefore involve multi-year and multi-location trials across different poplar varieties to validate the stability and broad applicability of *S. spiroverticillatus*-based control. Moreover, transcriptomic or proteomic approaches (*e.g.*, RNA-seq) could further elucidate the signaling pathways and defense genes activated by *S. spiroverticillatus* treatment, providing molecular insight into its biocontrol mechanisms.

## Conclusions

The fermentation broth of the biocontrol agent *Streptomyces spiroverticillatus* exhibited significant preventive and therapeutic efficacy against poplar canker. Treatment with the broth enhanced the innate disease resistance of poplar plants, as evidenced by increased levels of phenolic compounds, flavonoids, and lignin in leaves at various time points post-application. Furthermore, the activity of phenylalanine ammonia-lyase (PAL), a key enzyme in the phenylpropanoid pathway responsible for catalyzing the synthesis of these antimicrobial metabolites, was notably elevated. These results indicate that the fermentation broth effectively induces the accumulation of phenolic antifungal compounds in poplar, priming the plant’s chemical defenses prior to pathogen challenge and thereby strengthening its intrinsic capacity to resist fungal infection. In addition, treatment with *S. spiroverticillatus* fermentation broth enhanced chitinase activity in host plants, which is known to disrupt fungal cell wall integrity by hydrolyzing chitin. This response contributes to the suppression of pathogen proliferation and spread during infection.

## Supplemental Information

10.7717/peerj.20943/supp-1 Supplemental Information 1 The original measurement results The data for total phenols, flavonoids, lignin, chitinase, and PAL in the control and treatment groups from day 3 to day 21.

## Additional Information and Declarations

### Data Availability

The following information was supplied regarding data availability: The raw data are available in the Supplemental Files.
